# Supplementary material for: Therapeutic Potential of Deflamin against Colorectal Cancer Development and Progression
Source: Cancers (Basel). 2022 Dec 14;14(24):6182. doi: 10.3390/cancers14246182 (PMC9776913; doi:10.3390/cancers14246182)
Supplement: Supplementary file 1 [file cancers-14-06182-s001.zip › cancers-2053252-Supplementary.pdf]

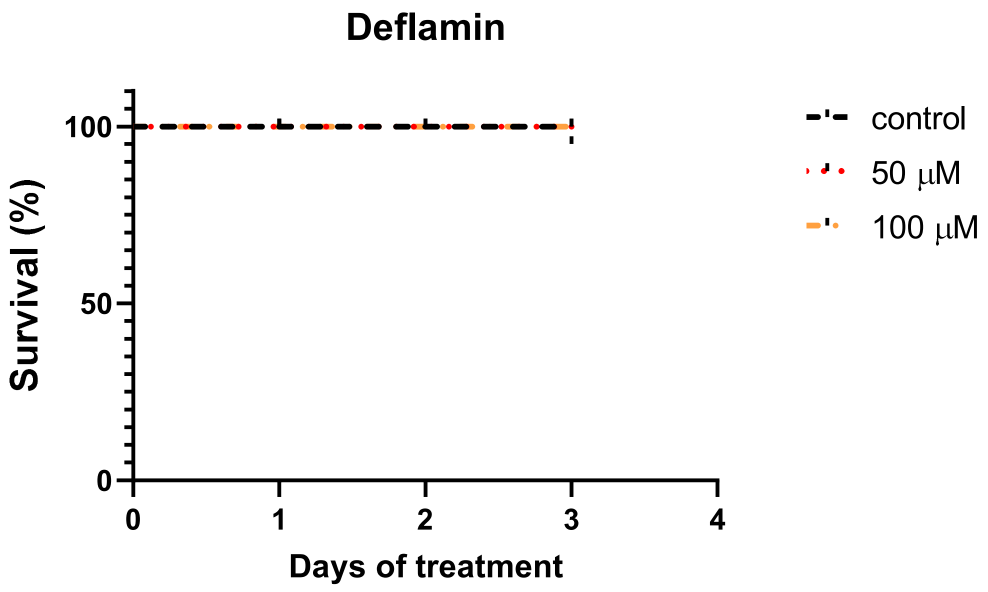

Supplementary Figure S1. Zebrafish lethality curves of the acute toxicity assay. At 72hpf zebrafish embryos were exposed to increased concentrations of deflamin (n=20) and mortality was evaluated during 72h of treatment.
